# Case Report: Hypomorphic Ligase 4 deficiency – a paradigm of immunodysregulation

**DOI:** 10.3389/fimmu.2025.1545630

**Published:** 2025-02-28

**Authors:** Catarina Andrade, Ana Isabel Cordeiro, Marta Valente Pinto, Conceição Neves, Catarina Martins, Jean-Pierre Villartay, João Farela Neves

**Affiliations:** ^1^ Primary Immunodeficiencies Unit, Hospital D Estefânia, Unidade Local de Saúde São José, Lisbon, Portugal; ^2^ Egas Moniz Center for Interdisciplinary Research (CiiEM), Egas Moniz School of Health and Science, Almada, Portugal; ^3^ Comprehensive Health Research Centre (CHRC), NOVA Medical School, Universidade NOVA de Lisboa, Lisbon, Portugal; ^4^ Imagine Institute, Hôpital Necker-Enfants Malades, Laboratory of Genome Dynamics in the Immune System, Institut National de la Santé et de la Recherche Médicale - Unité Mixte de Recherche (INSERM UMR1163), Paris, France

**Keywords:** case reports, inborn errors immunity, hypogammaglobulinemia, autoimmunity, V(D)J recombination, DNA damage repair

## Abstract

DNA Ligase 4 is critical to nonhomologous end joining, necessary for V(D)J recombination in T and B cell development. Ligase 4 deficiency is a rare autosomal recessive disorder caused by hypomorphic mutations in the DNA Ligase 4 gene, that can lead to a wide range of phenotypes. We describe a case of Ligase 4 deficiency causing a type of T-B-NK+ atypical SCID, highlighting the clinical and immunologic manifestations. An eight-year-old female, from São Nicolau Island (Cape Verde), presented at our hospital with a history of recurrent pneumonia and suppurative otitis, multiple skin lesions attributed to fungal and bacterial infections since the age of two, and recurrent diarrhea and growth impairment, beginning at the age of four. The laboratory workup showed almost absent B cells, marked hypogammaglobulinemia, and an impaired response to protein antigens. Flow cytometry revealed normal NK and T cell counts, but with nearly absent naïve T cells and TCR-Va7 expressing T lymphocytes, and reduced proliferative responses to mitogens and antigens. An oligoclonal Vβ repertoire was identified by FACS, and PROMIDISa analysis revealed a skewed TCRa repertoire signature. A 477 PID-related genes NGS panel identified a homozygous R278H mutation in the DNA Ligase 4 gene, previously reported to cause Ligase 4 deficiency. Immunoglobulin replacement and prophylactic therapies were started while waiting for hematopoietic stem cell transplantation. She has experienced fluctuating transaminase levels. The cutaneous biopsy was suggestive of lupus pernio. She has shown recurrent inflammatory signs in her limbs, with documented tenosynovitis on ultrasound. Homozygous R278H in Ligase 4 has been linked to various ranges of manifestations in Ligase 4 deficient patients. In our report, this genotype resulted in T-B-NK+ atypical SCID, that after proper prophylaxis has a predominant autoimmune phenotype.

## Introduction

Inborn errors of immunity (IEIs) are rare monogenic disorders characterized by a wide array of clinical manifestations, including infectious susceptibility, autoimmunity, autoinflammation, atopy, bone marrow defects, increased malignancy risk, or a combination of these (1).

DNA damage is a constant cellular obstacle, mitigated by highly conserved DNA repair mechanisms that preserve genomic integrity. Mutations in DNA replication, repair, or damage response pathways can cause syndromes with immunologic features, with DNA double-strand breaks (DNA-DSBs) being the most toxic lesions. Unrepaired or misrepaired DNA-DSBs can lead to tumorigenesis or cell death (2, 3). Certain cell types, such as lymphocytes, rely more heavily on DNA repair due to their high proliferation rates and specific recombination processes (3).

DNA Ligase 4 (LIG4) is critical to nonhomologous end joining, necessary for V(D)J recombination in T and B cell development. Both immunoglobulins (Ig) and T-cell receptors (TCR) are composed of heterodimers: Ig consists of heavy and light chains (κ or λ), while TCR is formed by α/β or γ/δ chains. The common basic structure of these receptors includes a Constant region and a Variable region, which is formed by combining Variable (V), Diversity (D), and Joining (J) gene segments, creating an extensive diversity of the immune repertoire (4).

**Figure 1:**
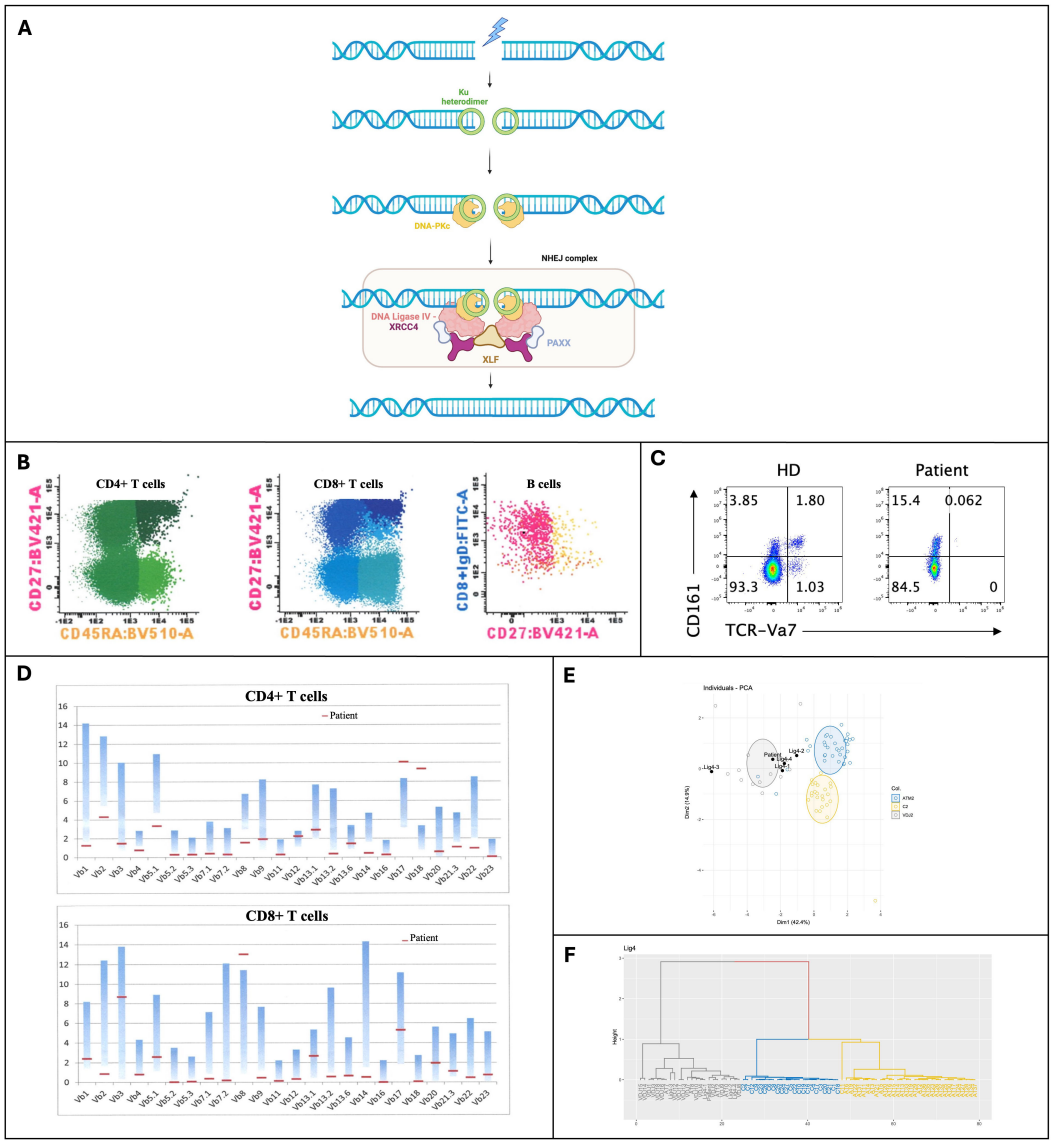
**(A)** Overview of the Non-Homologous End Joining (NHEJ) pathway for repairing DNA double-strand breaks, showing the recruitment and assembly of key proteins, including DNA Ligase 4 (Created in BioRender. Valente Pinto, M. (2025) https://BioRender.com/z21q491); **(B)** Immunophenotyping of peripheral blood by multiparametric flow cytometry; **(C)** Expression of TCR-Vα7 in T cells; **(D)** Repertoire in Fluorescence-Activated Cell Sorting (FACS) of CD4+ and CD8+ T cells; **(E, F)** TCRα repertoire analysis through PROMIDIS. BV421-A, Brilliant Violet 421-A; BV510-A, Brilliant Violet 510 fluorochrome; CD27, Costimulatory molecule; CD45RA, Marker of naïve T cells; CD161, Cluster of Differentiation 161; Dim, refers to the low expression; DNA-PKc, DNA-dependent Protein Kinase catalytic subunit; FITC-A, Fluorescein isothiocyanate; HD, Healthy Donor (control sample); Ku heterodimer, Protein complex; NHEJ, Non-Homologous End Joining; PAXX, Protein stabilizing the NHEJ repair complex; PCA, Principal Component Analysis; TCR-Vα7, T-cell receptor variable alpha chain 7; XLF, XRCC4-like factor; XRCC4, X-ray Repair Cross-Complementing Protein 4.

Among the three human DNA ligases, DNA LIG4 is the one that repairs DNA-DSBs through the non-homologous end joining (NHEJ) pathway. The repair of DNA-DSBs begins with the binding of the ring-shaped Ku heterodimer to the DNA ends, forming a Ku-DNA complex that recruits other NHEJ proteins such as DNA-PKcs and the DNA LIG4/XRCC4 complex, with PAXX and XLF as cofactors. During the formation of coding joints from RAG-initiated DNA-DSBs, the DNA ends are brought together by protein-protein interactions involving DNA-PKcs molecules. The nuclease Artemis, along with specialized DNA polymerases λ and μ and other enzymes, processes the DNA ends to ensure their compatibility (**Figure 1A**) (5, 6).

The DNA Ligase 4 gene, spanning 10.9 kb and consisting of two exons and one intron, is located on chromosome 13q33-q34. DNA LIG4 is a 911-amino acid protein with a core catalytic region that includes a DNA binding domain (DBD), a nucleotidyltransferase domain (NTD), and oligonucleotide/oligosaccharide-binding fold subdomains (OBD). The C-terminal region of DNA LIG4 contains two BRCT domains, a nuclear localization signal sequence, and a stretch of amino acids between the BRCT domains that mediate interaction with XRCC4 (7–9).

Through its crucial role in V(D)J recombination, DNA LIG4 not only contributes to receptor diversification but is also essential for the development of both B and T lymphocytes and for maintaining genome integrity during somatic rearrangement (4). Embryonic lethality has been documented in mice with a knockout of the DNA LIG4 gene. As expected, no cases of null mutations in the DNA Ligase 4 gene have been reported in humans (5, 10).

Since the 1990s, case reports have been published on patients with inherited mutant alleles of the DNA LIG4 gene that encode polypeptides with partial activity. In these cases, the mutated protein is severely compromised in its ability to form a stable enzyme-adenylate complex, although residual activity can be detected at high ATP concentrations (11).

LIG4 deficiency syndrome is an extremely rare disorder, with an incidence estimated to be around 1/1million, with around 120 cases reported in the literature (12). This autosomal recessive disease is caused by hypomorphic mutations that impair LIG4 function leading to deficient DNA damage repair mechanisms, and it is classified in the group of immunodeficiencies affecting cellular and humoral immunity, specifically T-B- Severe Combined Immunodeficiency (SCID), according to the 2024 Update on the Classification of Human Inborn Errors of Immunity (8, 13, 14).

LIG4 deficiency typically presents with congenital microcephaly, atypical facial features, growth failure, developmental delay, combined immunodeficiency, sensitivity to ionizing radiation, and increased susceptibility to malignancy. Nevertheless, this syndrome has been progressively recognized as displaying a broad spectrum of phenotypes, which can be attributed to redundant DNA repair mechanisms and the residual activity of LIG4. This partial functionality may retain some recombination activity, allowing for the development of a rudimentary immune system (10, 15).

In this report, we describe a case of Ligase 4 deficiency, characterized by T-B-NK+ leaky SCID, presenting with recurrent infection, tenosynovitis and lupus pernio.

The legal representative of the child patient provided informed consent for this report and for the publication of photographs of the clinical features.

## Case description

An eight-year-old female, originary from Cape Verde, with no relevant family history, was referred to the Primary Immunodeficiency (PID) Unit. She was born at term, with no complications during pregnancy, the neonatal period, or early childhood, and an unremarkable history after live vaccines.

She presented a history of recurrent pneumonia and suppurative otitis, multiple skin lesions attributed to fungal and bacterial infections since the age of 2 years-old, and recurrent diarrhea beginning at the age of four. She was hospitalized repeated times and was administered extended courses of antibiotics. Her condition progressed with failure to thrive, although at physical examination she did not present any signs of microcephaly, dysmorphisms, or developmental delay. Upon our admission, notable findings included a malnourished appearance (weight at the 3rd percentile; height at the 30th percentile; body mass index at the 1st percentile), scattered crackles on bilateral lung auscultation, an ulcerated lesion on the scalp, healing cutaneous lesions on the limbs, and tympanosclerosis and bilateral perforations. No lymphadenopathy or organomegaly were identified.

The laboratory workup revealed marked hypogammaglobulinemia and impaired vaccine responses to protein antigens (diphtheria and tetanus). Immunophenotyping of peripheral blood performed with multiparametric flow cytometry showed absent B cells, with normal T and NK cell counts. However, naïve CD4 and CD8 T cells were almost absent (**Table 1**, **Figure 1B**). Moreover, we noticed an absence of TCR-Va7 expressing T cells (**Figure 1C**), suggesting an impaired V(D)J recombination and/or DNA repair in this patient (16). An oligoclonal TCR Vβ repertoire was identified by Fluorescence-Activated Cell Sorting (FACS) (**Figure 1D**). Moreover, analysis of the TCRa repertoire by PROMIDISa highlighted a typical signature of V(D)J recombination defects, as observed in four other LIG4 deficiency patients (**Figures 1E, F**) (16). Functional assays showed reduced proliferative responses to mitogens and antigens (**Table 1**). A panel of 477 PID-related genes was investigated by Next-generation sequencing (NGS) and identified (confirmed by Sanger sequencing) a homozygous R278H mutation in the LIG4 gene, inherited from the heterozygous parents, who have no known history of autoimmunity or immune dysregulation (immune-phenotyping was not performed). This mutation has previously been reported to cause LIG4 deficiency.

**Table 1:** Immunologic study results.

Parameters	Results (Reference)
Hemoglobin	13.6 g/dL (11.5-15.5)
Leukocytes	3710 cells/µl (5000-13000)
Neutrophils	1680 cells/µl (2000-8000)
Lymphocytes	1510 cells/µl (1100-5900)
Eosinophils	100 cells/µl (100-1000)
Basophils	20 cells/µl (0.0-100)
Monocytes	400 cells/µl (100-1000)
Platelets	208000 cells/µl (180000-400000)
Reticulocytes	2.65% (0.5-2.5)
IgG	0.81 g/L (5.93-17.30)
IgA	<0.26 g/L (0.33-3.60)
IgM	<0.18 g/L (0.55-1.6)
IgE	< 2.0 KUI/L (0-90)
IgG anti-diphtheria	<0.01 IU/mL (<0.10 - no vaccine response)
IgG anti-tetanus	<0.01 IU/mL (<0.10 - no vaccine response)
B cells^*^	0.24%; 5.9 cells/µl (6.1-25.2%; 175-1191)
Pregerminal	77.97%; 4.6 cells/µl (47.8-88.4%; 107-970)
Postgerminal	23.73%; 1.4 cells/µl (8.1-33.3%-44.2-312)
Unswitched	12.71%; 0.75 cells/µl (3.1-22.0%; 22.2-178)
Switched	10.34%; 0.61 cells/µl (2.0-17.4%; 25.4-175)
T cells^*^	88.26%; 2188 cells/µl (55-97%; 1297-4202)
CD4+CD8-	43.97%; 1090 cells/µl (26-61%; 663-2559)
Naïve	1.35%; 14.7 cells/µl (42-99%; 316-2037)
Central memory	33.21%; 362 cells/µl (0.35-100%; 218-608)
Effector memory	64.04%; 698 cells/µl (0.27-18%; 10.6-96.2)
Effector TD	1.41%; 15.4 cells/µl (0.0031-1.8%; 0-43.8)
CD8+CD4-	22.71%; 563 cells/µl (13-47%; 310-1384)
Naïve	4.16%; 23.4 cells/µl (16-100%; 163-886)
Central memory	29.66%; 167 cells/µl (1-38.9%; 60.4-365)
Effector memory	38.72%; 218 cells/µl (0.4-100%; 1.6-78.8)
EffectorTD CD27+d	0.034%; 1.9 cells/µl (0.04-1.8%; 2.2-176)
EffectorTD CD27-	2.7%; 153 cells/µl (0.13-4.6%; 0.81-358)
CD4-CD8-/dim TCRgd+	23.77%; 520 cells/µl (2-24%; 63.2-560)
CD4-CD8-/dim TCRgd-	0.26%; 14.7 cells/µl (0.2-0.95%; 13.2-79.8)
NK cells	11.5%; 285 cells/µl (2-31%; 116-1241)
Response to Phytohemagglutinin (PHA)	6.67% (Control 33.3%)
Response to Purified Protein Derivative (PPD)	2.08% (Control 3.83%
Response to Candida albicans	1.16% (Control 6.51%)
Response to Tetanus Toxoid (TT)	1.09% (Control 4.86%)

^*^The markers used for each population of T and B cells are those established by the EuroFlow Consortium regarding the Primary Immunodeficiency Orientation Tube (PIDOT, Cytognos, Salamanca, Spain) (33).

IgA, Immunoglobulin A; IgE, Immunoglobulin E; IgG, Immunoglobulin G; IgM, Immunoglobulin M; Effector TD, Terminally Differentiated Effector cells; NK cells, Natural Killer cells.

Subcutaneous immunoglobulin replacement and prophylactic itraconazole and cotrimoxazole were started while waiting for hematopoietic stem cell transplantation (HSCT).

Since admission, she has maintained elevated liver enzyme levels (alanine aminotransferase (ALT), aspartate aminotransferase (AST), alkaline phosphatase, gamma-glutamyl transpeptidase), in a fluctuating pattern, with normal bilirubin level and normal tests of hepatic synthetic function, without clinical signs of chronic hepatic disease (**Table 2**). The analytical investigation of the hepatitis did not identify any etiology, including multiple stool analyses such as bacteriological and mycological cultures, testing for ova and parasites, toxin screening, molecular viral detection, and PCR for *Cryptosporidium*. Transabdominal ultrasonography with Doppler revealed a normally sized liver with regular contour and homogeneous echotexture, non-dilated gallbladder and bile ducts, normally sized pancreas, and no alterations in the hepatic and portal vessels (**Table 2**). Currently, she is awaiting a liver biopsy.

**Table 2:** Results of analytical investigation conducted for hepatitis.

Parameters	Results (Reference)
Liver test - maximum value	
ALT	132 U/L (<19)
AST	236 U/L (<33)
Gamma-glutamyl transpeptidase	427 U/L (4.0-12.0)
Alkaline phosphatase	815 U/L (142 – 335)
Total Bilirubin	0.26 mg/dl (< 0.50)
Infections	
Hepatitis B, Hepatitis C, and HIV 1 and 2 viral loads	Negative
PCR of VZV, HSV1, HSV2, HHV6, HHV7, EBV, CMV, Parvovirus B19, Enterovirus, Adenovirus, and Toxoplasma gondii	Negative
Stool parasitology exam for ova and larvae	Negative
Stool test for Adenovirus, Astrovirus, Cryptosporidium, and Norovirus	Negative
IGRA T-Spot	Negative
Autoimmunity	
ANA, anti-dsDNA, ENAs, Rheumatoid Factor, anti-Beta2GP1, anti-cardiolipin, anti-gliadin, anti-transglutaminase, anti-MPO, anti-PR3, anti-GBM, anti-AMA-M2, anti-ASMA, anti-LKM1, anti-Citosol 1, anti-gp210, anti-SLA/LP, anti-Sp100, Direct Coombs Test	Negative
Other causes	
Ceruloplasmin level	0.49 g/L (0.20 – 0.60)
Serum creatine kinase	94 U/L (<170)
Ammonia	101 ug/dL (27.2 – 102)
Thyroid-stimulating hormone	1.70 uUI/mL (0.60 – 4.84)
Thyroxine	1.30 ng/dL (0.97 – 1.67)

ALT, alanine aminotransferase; ANA, Antinuclear Antibodies; anti-AMA-M2, anti-mitochondrial antibodies M2; anti-ASMA, anti-Smooth Muscle antibody; anti-Beta2GP1, anti-beta-2-glycoprotein 1 antibodies; anti-dsDNA, anti-double-stranded DNA antibodies; anti-GBM, anti-glomerular basement membrane; anti-gp210 -anti-glycoprotein gp210 antibody; anti-LKM1, Liver Kidney Microsomal type 1 antibodies; anti-MPO, anti-neutrophil cytoplasmic antibodies; anti-PR3, anti-neutrophil cytoplasmic antibodies; anti-SLA/LP, anti-soluble liver antigen/liver pancreas antibodies; anti-Sp100, anti-Sp100 nuclear antigen; AST, aspartate aminotransferase; CMV, Cytomegalovirus; EBV, Epstein-Barr virus; ENAs, Extractable Nuclear Antigens; HHV, Human Herpesvirus 6 and 7; HIV, Human Immunodeficiency Virus 1 and 2; HSV, Herpes simplex virus 1 and 2; IGRA – Interferon-Gamma Release Assay; PCR, Polymerase Chain Reaction; VZV, Varicella-Zoster virus.

CT scan of the thorax identified bronchiectasis in the middle and lower lobes of the right lung. Pulmonary function tests demonstrated adequate alveolar-capillary transfer of carbon monoxide.

**Figure 2:**
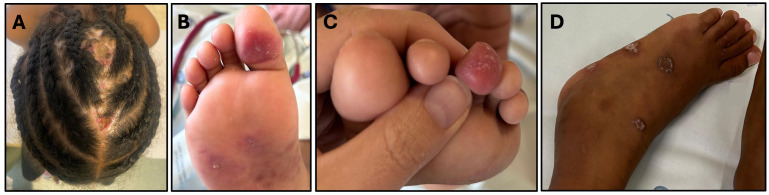
Clinical features: **(A)** Ulcerated lesion on the scalp; **(B, C)** Violaceous lesions suggestive of lupus pernio on the plantar region and toes were characterized histopathologically by a lymphocytic perivascular and periannexal inflammatory infiltrate with associated telangiectasias. CD123 immunohistochemistry revealed a mild increase in plasmacytoid dendritic cells; **(D)** Signs of tenosynovitis in the tibiotarsal region.

She has recurrent cutaneous lesions that evolved with hyperpigmented scar lesions on the limbs, with persistent alopecia in the occipital region of the scalp, characterized by lesions with a hyperkeratotic border and an atrophic center with areas of scaling and crusting (**Figure 2A**). She also presents with erythematous-violaceous papules on the plantar regions and lateral borders of the feet, affecting the fingers and toes, whose biopsy didn’t identify granulomas but a dermic, perivascular, both superficial and deep lymphocytic infiltrate, suggestive of lupus pernio (**Figures 2B, C**). An extensive investigation failed to identify any micro-organism, namely Mycobacteria (via culture and PCR), as well as vaccine strain rubella, Helicobacter, Mycoplasma, Ureaplasma, or other atypical pathogens through molecular determination (PCR), with culture-independent approaches being even more critical in immunocompromised patients and in cases involving fastidious organisms (17).

She has experienced recurrent inflammatory signs in her limbs, with edema and pain in her wrists, back of both hands and left tibiotarsal region, with limping and impact on daily activities, with documented tenosynovitis on ultrasound (**Figure 2D**). This was accompanied by an elevation in erythrocyte sedimentation rate, without impact on C-reactive protein (ESR 56mm/h, CRP 3.9mg/L). She started therapy with non-steroidal anti-inflammatory drugs with dramatic clinical improvement, supporting the inflammatory cause of the tenosynovitis.

Since the beginning of the immunoglobulin replacement therapy and antimicrobial prophylaxis, there has been no record of bacterial infections. On the other hand, she has asymptomatic, mild, intermittent Cytomegalovirus (CMV) viremia (maximum viral load of 116.0 IU/mL).

## Discussion

The rising number of LIG4 deficiency cases reported in the literature underscores the wide clinical spectrum, even among patients with similar genotypes (13, 18).

Our case involves a female patient, consistent with the 68% female prevalence reported in a series of 41 cases described by Staines Boone et al (18). The clinical presentation began at two years of age, and the diagnosis was made at eight years of age. The median age at diagnosis in the aforementioned series was nine years, ranging from 0 to 48 years (18). Different degrees of partial activity of LIG4 can justify the wide range of presentation ages, and the development of a residual immune system can account for later manifestations. An *in vivo* plasmid-rejoining assay demonstrated that Non-Homologous End Joining was impaired but not completely eliminated in cells expressing the R287H mutant protein (10, 11, 18). The delay in the diagnosis can be partially attributed to the evacuation from the country of origin to a reference center in another country.

The initial clinical presentation was marked by susceptibility to infection, with multiple alarm signs suggestive of an inborn error of immunity, but the absence of microcephaly or syndromic features, which are classic signs of LIG4 deficiency, could have also contributed to a delayed referral and consequently diagnosis. The literature indicates that the immune system can range from normal to severely compromised, with the onset of symptoms occurring from early infancy to later in life (18). In this case, the analytical studies were consistent with of T-B-NK+ leaky SCID, as the following criteria were met: less than 20% of CD4+ T cells are naïve, absence of TCR-Va7 expressing T lymphocytes, impaired PROMIDISa signature, presence of oligoclonal T cells, reduced proliferation, and detection of a pathogenic gene variant (19). Cumulatively, she presented with B lymphopenia and hypogammaglobulinemia.

The R278H mutation was the first mutation in the LIG4 gene reported in humans. It was identified in a case of a patient with leukemia who developed an overresponse to radiotherapy associated with increased cellular radiosensitivity (8). It is a missense mutation resulting from the substitution of histidine for arginine within a motif close to the active site lysine that is highly conserved among ATP-dependent DNA ligases (20, 21). Consequently, adenylate complex formation and adenylation activity are impaired but not abolished. Mutated cells display V(D)J recombination with decreased precision in signal joint formation (20). Rucci et al. generated a knock-in murine model carrying a homozygous R278H mutation and documented that peripheral T lymphocytes show an anergic phenotype, reduced viability, and a restricted repertoire, compatible with human leaky SCID (22). Park et al., using a knock-in murine model, documented that the R278H mutation impairs B-lymphopoiesis. This mutation results in reduced class switch recombination efficiency, high turnover of the LIG4 protein, defective proliferation, increased chromosomal breaks, and apoptosis, primarily due to error-prone NHEJ (21). Our case highlights the phenotypic variability inherent to LIG4 deficiencies, even among individuals with similar genotypes. Notably, it differs from two cases previously reported by our team, both with a homozygous missense R278H mutation (13). These cases presented with hypopigmented lesions, no facial dysmorphisms, microcephaly, or neurodevelopmental abnormalities, as well as B-cell lymphopenia and hypogammaglobulinemia, with no clinical or laboratory evidence of autoimmunity. The cases also differed in their ages of presentation (16 years vs. 3 years), with the degree of immunological impairment being more severe and infection susceptibility more pronounced in the older individual, whose symptoms manifested during adolescence (13).

Autoimmunity may also be a manifestation of inborn errors of immunity, with the prevalence of autoimmune/inflammatory diseases increasing with age in primary immunodeficiency cohorts (23). Autoimmunity occurs frequently in T-cell immunodeficiencies, including leaky severe combined immunodeficiency, with more than 50% of patients exhibiting an autoimmune component (24). Various molecular mechanisms can affect multiple immune pathways. These include absolute lymphopenia causing a lack of regulatory lymphocytes, apoptosis defects, loss of central tolerance, loss of peripheral tolerance, alterations in inhibitory signaling and gain of signaling function, unregulated type 1 interferon responses, and complement defects impairing the removal of immune complexes and cell debris (23, 24). A very restricted Vbeta repertoire and sensitivity to radiation has been linked to homozygous R278H mutation, which can contribute to the loss to peripheral tolerance and potentially leading to immunedysregulation. Interestingly, LIG4 missense mutations leading to haploinsufficiency have recently been described to underlie autoimmunity in heterozygous LIG4 deficiency. These patients present a dominantly inherited familial immune-dysregulation, with autoimmune cytopenias, lymphoproliferation, hypogammaglobulinemia and recurrent infections. This stresses the importance of LIG4 to the maintenance of a proper immune homeostasis (12).

In our case, it seems that after proper prophylaxis and immunoglobulin replacement for infection control, an immunodysregulatory/inflammatory phenotype predominates. The elevation in transaminases preceded the initiation of hepatotoxic drugs. The liver biopsy, currently pending, will be critical for determining the etiology of hepatitis, including ruling out secondary causes such as infectious etiologies. It is noteworthy that case series in the literature report liver involvement with jaundice, hepatomegaly, and sclerosing cholangitis in patients with LIG4 deficiency (18).

Although DNA breaks can trigger an immune response, the exact mechanism behind autoimmune disease development remains unclear. Silva et al. found that LIG4 polymorphisms did not show a statistically significant increased risk for systemic lupus erythematosus (SLE) clinical features, except for a trend towards cutaneous alterations (25). Our patient met the diagnostic criteria for pernio, characterized by localized erythema and swelling in acral sites, persisting for more than 24 hours, with histopathologic findings from a skin biopsy consistent with pernio. In a case series of 104 patients, 4% had connective tissue disease (non-lupus), and 3% had an associated hematologic malignancy. Although the majority of patients with pernio in the aforementioned series did not have an underlying systemic association, our case suggested a possible link between inflammatory skin lesions and primary immunodeficiencies (26). In the literature, mucocutaneous manifestations of LIG4 deficiency are varied, including psoriasis, telangiectasias, rash, erythema, pallor, cutis marmorata, single palmar crease, hypopigmentation, and stomatitis (13, 18).

Similarly, musculoskeletal involvement with clinical and imaging signs of inflammation compatible with tenosynovitis, supported by an inflammatory pattern in analytical tests, also indicates a condition of inflammation/autoimmunity. In fact, rheumatological diseases such as arthritis are not uncommon complications of inborn errors of immunity, particularly in primary immune regulatory disorders. Monogenic disorders can even mimic juvenile idiopathic arthritis. Consequently, longitudinal clinical phenotyping is essential. Recognizing that single gene diagnoses can have significant implications for the prognosis and management of autoimmune disease is crucial (24, 27).

Patients with combined immunodeficiencies are extremely susceptible to CMV infection. Al-Herz et al. documented an overall viral infection rate of 31.7% in 274 children with primary immunodeficiencies, predominantly those affecting cellular and humoral immunity, with CMV accounting for 22.2% of these infections (28). Managing CMV infection in these patients is challenging due to the risks of drug-related toxicity and antiviral resistance. CMV is a significant risk factor for morbidity and mortality before and after hematopoietic stem cell transplantation. Effective management includes preventing and promptly controlling CMV infection, coupled with early HSCT or gene therapy, which are crucial for improving outcomes in children with inborn errors of immunity (29).

HSCT could be a curative treatment option. The choice to proceed with a transplant is a personalized decision that depends on various factors, including the intensity of clinical symptoms, the degree of T-cell deficiency, the policies of the medical center, donor availability, and the preferences of the patient’s family (30). Given the rarity of DNA LIG4 deficiency and the limited number of patients undergoing HSCT, no standardized conditioning regimen has been established. Due to the severe radiosensitivity of these patients, irradiation should be excluded from the conditioning protocol (31). The increased toxicity in individuals with DNA repair disorders imposes significant limitations on chemotherapy (32). In our case, in addition to these challenges, social conditions such as evacuation from the country of origin without family support, except for the mother, language barriers, low economic resources, and illiteracy also pose significant constraints.

In conclusion, this case of Ligase 4 deficiency illustrates the complex interplay between infection susceptibility and autoimmunity, emphasizing the importance of genetic diagnosis given the clinical heterogeneity. The patient’s evolution highlights the limitations of current management strategies and the urgency for hematopoietic stem cell transplantation, which is constrained by the lack of a standardized conditioning regimen and social factors. This underscores the need for a holistic approach to care and the challenges of managing a rare immunodeficiency. This case enriches the literature on Ligase 4 deficiency and manifestations spectrum and reinforces the importance of early diagnosis and the use of tailored treatment strategies in improving patient outcome.

## Data Availability

The original contributions presented in the study are included in the article/supplementary material. Further inquiries can be directed to the corresponding author.
